# Optimizing Students’ Mental Health and Academic Performance: AI-Enhanced Life Crafting

**DOI:** 10.3389/fpsyg.2020.01063

**Published:** 2020-06-03

**Authors:** Izaak Dekker, Elisabeth M. De Jong, Michaéla C. Schippers, Monique De Bruijn-Smolders, Andreas Alexiou, Bas Giesbers

**Affiliations:** ^1^Department of Technology and Operations Management, Rotterdam School of Management, Erasmus University, Rotterdam, Netherlands; ^2^Research Centre Urban Talent, Rotterdam University of Applied Sciences, Rotterdam, Netherlands; ^3^Department of Management, Tilburg School of Economics and Management, Tilburg University, Tilburg, Netherlands; ^4^Information Management and Consulting, Rotterdam School of Management, Erasmus University, Rotterdam, Netherlands

**Keywords:** life crafting, chatbot, mental health, academic performance, academic success, academic achievement, goal setting, well-being

## Abstract

One in three university students experiences mental health problems during their study. A similar percentage leaves higher education without obtaining the degree for which they enrolled. Research suggests that both mental health problems and academic underperformance could be caused by students lacking control and purpose while they are adjusting to tertiary education. Currently, universities are not designed to cater to all the personal needs and mental health problems of large numbers of students at the start of their studies. Within the literature aimed at preventing mental health problems among students (e.g., anxiety or depression), digital forms of therapy recently have been suggested as potentially scalable solutions to address these problems. Integrative psychological artificial intelligence (AI) in the form of a chatbot, for example, shows great potential as an evidence-based solution. At the same time, within the literature aimed at improving academic performance, the online life-crafting intervention in which students write about values and passions, goals, and goal-attainment plans has shown to improve the academic performance and retention rates of students. Because the life-crafting intervention is delivered through the curriculum and doesn’t bear the stigma that is associated with therapy, it can reach larger populations of students. But life-crafting lacks the means for follow-up or the interactiveness that online AI-guided therapy can offer. In this narrative review, we propose to integrate the current literature on chatbot interventions aimed at the mental health of students with research about a life-crafting intervention that uses an inclusive curriculum-wide approach. When a chatbot asks students to prioritize both academic as well as social and health-related goals and provides personalized follow-up coaching, this can prevent -often interrelated- academic and mental health problems. Right on-time delivery, and personalized follow-up questions enhance the effects of both -originally separated- intervention types. Research on this new combination of interventions should use design principles that increase user-friendliness and monitor the technology acceptance of its participants.

## Introduction

One in three students leaves higher education without attaining the higher education degree for which they enrolled (Organisation for Economic Co-operation and Development (OECD), 2010, 2013, 2019). Research suggests that students are having trouble adjusting to tertiary education, leading them to underperform academically (Perry, 1991). For example, students are said to have problems with integrating academically and socially (Tinto, 1998, 1999) and with managing their learning processes (e.g., goal setting, planning, monitoring, and time management; Robbins et al., 2004; Richardson et al., 2012). Not only does the first year of college come with a relatively high risk of not succeeding academically, it also coincides with a higher risk of mental health-related issues and subsequently low levels of well-being (Hunt and Eisenberg, 2010; Auerbach et al., 2018; Bruffaerts et al., 2018; Choi, 2018). Mental health and well-being are related and contribute to the decrease of students’ academic performance (in the current study defined as student retention, grade point average and obtained credits Bruffaerts et al., 2018). College students with mental health problems are twice as likely to drop out (Kessler et al., 1995; Hartley, 2010), and depression and suicidal thoughts relate to a lower GPA (Mortier et al., 2015; De Luca et al., 2016). Mental health and academic performance are thus interrelated.

Underlying both mental health and academic performance is a broader conception of ‘eudaimonic’ well-being as self-realization and meaning (Waterman, 1993; Ryan and Deci, 2001). Research suggests that undergraduate students often have difficulty with finding meaning (Steger et al., 2008) or a clear sense of purpose or direction in life (Schippers and Ziegler, 2019). However, having self-concordant goals (i.e., goals that align with one’s values and passions), relates to higher academic performance (Sheldon and Houser-Marko, 2001), higher subjective well-being (Sheldon, 2002), and lower symptoms of depression (Sheldon and Kasser, 1998).

From this point of view, Schippers and Ziegler (2019) suggested using life-crafting interventions in order to help students reflect on their salient personal goals and improve their academic performance and well-being. Life crafting is a combination of techniques that allows people to (1) find their values and passions using expressive writing, (2) contrast desired habits and domains of life with the current state using mental contrasting, (3) use goal setting to prioritize ambitions and guide effort, and (4) effectuate their plans using implementation intentions. Thus, it helps people to become more specific about their goals and goal achievement plans (GAP). Together the exercises lead to a process of life crafting, defined as:

A process in which people actively reflect on their present and future life, set goals for important areas of life—social, career, and leisure time—and, if required, make concrete plans and undertake actions to change these areas in a way that is more congruent with their values and wishes. (Schippers and Ziegler, 2019, p. 3).

The potential impact of life-crafting interventions seems promising, particularly because it is online and, therefore, scalable. However, it also has three weaknesses. First off, the current intervention technique does not ask follow-up questions. When students write brief answers to the life-crafting questions, the online questionnaire is not programmed to encourage the students to explicate their thoughts further and write more. A second shortcoming regards the methods for follow-up. Students who participated in the life-crafting exercises suggested that the intervention would improve if the intervention includes personal guidance after the initial phase. The email reminders used so far were not interactive or personalized. Thirdly, the current program does not differentiate for individual needs. For students without problems or with minor problems, the life-crafting program might be enough to boost their academic performance and well-being. However, others might require more follow-up and interaction, or might need coaching on mental health problems that interfere with their academic performance. Coaches and psychologists could facilitate personalized follow-up and interaction, but it would be time-consuming and costly. Most higher education institutions do not have the capacity to offer this kind of support. Therefore, there is a need for other scalable solutions, that offer a personalized and interactive program and contribute to early recognition of problems with academic performance or well-being, in order to prevent more severe problems.

A contemporary solution that is gaining momentum in the mental health-care sector is a mental-health chatbot (Provoost et al., 2017; Abd-alrazaq et al., 2019; Vaidyam et al., 2019). A chatbot is a computer program designed to simulate human conversation and is able to create the illusion of intelligent conversation (Warwick and Shah, 2014; Abdul-Kader and Woods, 2015) (for a review, see Fulmer, 2019). In a university setting, chatbots are predominantly used to provide cognitive behavioral therapy (Fitzpatrick et al., 2017; Fulmer et al., 2018; for an overview see Lattie et al., 2019). Other potential positive effects (e.g., on academic performance or well-being) have not yet been studied. Although in general chatbots show promising results (Provoost et al., 2017; Lattie et al., 2019), they are focused on offering therapy, and individuals may not use a health care service due to fears of stigma (Clement et al., 2015; Stewart et al., 2019). To illustrate: fewer than half of the college students who report suffering from one or more mental disorders seek treatment for those problems (Zivin et al., 2009; Auerbach et al., 2018; Stewart et al., 2019). Furthermore, the majority of students will probably not require cognitive behavioral therapy but would benefit from individualized coaching to overcome the problems they face during the transition to tertiary education. Therefore, in this narrative review, we propose to combine the two lines of research and to deliver a life-crafting intervention through an interactive chatbot. The chatbot can stimulate students to elaborate their answers to the life-crafting intervention, offer interactive and personalized follow-up, and also mental health coaching if needed.

Several studies (e.g., Tinto, 1975, 1998, 1999) indicate that both the transition to tertiary education as well as processes underlying student attrition never occur in isolation, but are the result of a longitudinal process of interrelated individual and environmental factors. We, therefore, advocate a holistic approach that stimulates students to steer their academic work, their social life, and health in the right direction. This proposed life-crafting method offers a positive approach aimed at improvement instead of a more narrow problem-centered approach toward remediation of mental health problems in student populations (Schippers and Ziegler, 2019). Therefore, the intervention can be targeted at all first-year students instead of a group of identified at-risk students, which lowers the threshold to engage with the intervention and avoids stigma.

Below, we first provide more background information about the mental health and well-being of students and how this relates to academic performance. Subsequently, to provide a rationale for combining a life-crafting intervention with a mental health chatbot, we will first outline what a life-crafting intervention looks like, and then focus on describing in more detail current internet-based mental health care and especially mental health-care chatbots. After that, we describe how we propose to integrate life crafting into an AI-enhanced mental health chatbot. Finally, we present a conceptual model and guidelines for future research to examine the effectiveness of the proposed intervention.

## Mental Health, Well-Being and Academic Performance

Generally speaking, mental health problems have a high prevalence among students in higher education. One in three college students reports one or more mental health problems (Hunt and Eisenberg, 2010; Auerbach et al., 2018; Bruffaerts et al., 2018). According to a recent study, including students attending 19 colleges across eight countries (*N* = 13,984), depression disorders are most common, followed by generalized anxiety disorders (Auerbach et al., 2018). At this moment, worldwide, roughly 70% of high school graduates attend college (Auerbach et al., 2018; Bruffaerts et al., 2018). The college years are a peak period for the onset of many common mental disorders, particularly mood, anxiety, and substance use disorders (De Girolamo et al., 2012; Ibrahim et al., 2013).

Part of these problems can be explained by ‘study stress’ and academic underperformance. Having to study and perform under pressure in college is found to correlate with anxiety and lower well-being (Centre for Education Statistics and Evaluation, 2015; Cant, 2018). Procrastinating and underperforming in college have been found to predict depression, low self-esteem, and anxiety (Saddler and Sacks, 1993; van Eerde and Klingsieck, 2018). Simultaneously, mental health-related issues influence academic performance (Kessler et al., 1995; Steel et al., 2001; Hartley, 2010; Kim and Seo, 2015; Bruffaerts et al., 2018). There is, as such, an interrelatedness between academic performance and mental health issues. In order to understand this interrelatedness, and propose solutions that do not improve one at the cost of the other, we should clarify two different underlying conceptions of well-being.

The symptoms of mental health issues are mostly coined in terms of negative affect: feelings of pain, stress, depletion. The absence of negative affect, in combination with positive affect (feelings of pleasure and joy), determines one’s subjective (or ‘hedonic’) well-being (Kahneman, 1999). In itself, this hedonic perspective on well-being can be a bad indicator of healthy living, given that it can lead to a focus on symptoms only or shortcuts (Ryff and Singer, 2008). A lifestyle aimed solely at hedonic well-being is more likely to be detrimental to well-being in the long run (Huppert et al., 2004; Anić and Tončić, 2013; Baumeister et al., 2013). More specifically, pursuing hedonic well-being can conflict with academic and career success, given that studying or working is not always fun and can require hard and arduous work.

Contrary to the hedonic view on well-being, the ‘eudaimonic’ view on well-being, states that well-being is attained when people live according to their most deeply felt values and are holistically engaged (Waterman, 1993). Both types of well-being are overlapping, yet distinct, and correlate moderately (Compton et al., 1996). Ryan and Deci (2001) argue that obtaining the basic needs (competence, relatedness, and autonomy) improves both hedonic as well as eudaimonic well-being. Lacking one or more of these needs, on the other hand, decreases both types of well-being.

When students attend college, they make the transition from late adolescence to emerging adulthood. Emerging adulthood (ages 18–29 years) is a developmentally crucial period that can be defined by shifts in autonomy (e.g., leaving the home, being expected to organize self-study), relational instability, and shifts in expected competence (Burris et al., 2009; Evans et al., 2009; Auerbach et al., 2018; Bruffaerts et al., 2018). This could explain why this period, and the first year of university, in particular, involves such a high rate of dropout and academic underperformance. Interventions that aid students in their shifts in autonomy, relatedness, and competence could thus be of particular value at the start of the study.

## Life Crafting

As a method of improving both the academic performance of students and their well-being, Schippers and Ziegler proposed using a ‘life-crafting’ intervention. The online life-crafting intervention consists of several integrated components. These components build on a range of empirically tested mechanisms that aid its participants to reflect on the present and future life, set goals and make plans and undertake actions in a way that is congruent with their values (Schippers and Ziegler, 2019).

The first stage of the intervention guides participants through the process of finding a self-concordant passion or purpose. This phase is not merely aimed at understanding what one likes or enjoys (hedonic well-being), but about finding out what one values as relevant and meaningful. Similar to the Japanese concept of ‘Ikigai’; a reason for being (Sone et al., 2008), and eudaimonic well-being, which includes meaning and self-realization (Ryan and Deci, 2001). This purpose is self-concordant when it is both intrinsically as well as extrinsically worth pursuing (Sheldon and Houser-Marko, 2001; Sheldon, 2002). The exercises stimulate participants to choose goals that the person truly believes to be important. This improves the chance that one’s (goal pursuing) actions are in accordance with one’s values.

Secondly, the planning phase involves ranking goals and mental contrasting (Oettingen, 2000, 2012). This phase helps students to formulate how their desired future differs from their current state. Participants contrast their imagined best possible outcome that is related to the goal with an inner obstacle that stands in the way. This technique is applied to competencies, habits, social life, career, and health. Questions direct the students to describe what competencies and habits they already possess and which desired and needed competencies and habits they lack. Merely thinking about an ideal future can lead to positive affect but decreases the chances that a person takes action in order to realize the desired future (Oettingen and Sevincer, 2018). Contrasting the ideal future with the current state, on the other hand, leads to more effort and positive outcomes (Oettingen et al., 2010; Oettingen, 2012). Knowing which habits one would like to change, improves the chances of actual behavioral change (Holland et al., 2006; Graybiel and Smith, 2014). With the use of a goal attainment plan (GAP), participants can bridge this gap (Schippers and Ziegler, 2019). The same questions are then applied both on their social life, their career/study, and their health.

Thirdly, participants use the goal-setting technique to formulate and prioritize their most important goals. They are encouraged to balance and prioritize social, career, and health-related goals. By doing so, they are stimulated to develop harmonious passion instead of obsessive work passion, which is related to conflicts between different domains of life (Curran et al., 2015). When writing their goals, they are asked to formulate ambitious but specific and attainable goals. This is a technique which is developed by Locke and Latham. Goal setting directs energy to the goal-related actions and improves self-regulated learning and motivation. Prior research has shown that writing about passions and goals is related to increased academic performance (Morisano et al., 2010; Schippers et al., 2015, 2020). Although it matters whether these are grade goals or task goals (Clark et al., 2019), and reflective goal setting has shown both positive (Morisano et al., 2010; Schippers et al., 2015, 2020) as well as no results (Dobronyi et al., 2019).

Finally, as part of the life-crafting process, participants design implementation intentions they require to execute their plans. Implementation intentions are ‘if-then’ plans which aid the person in making goal-related choices in a clutch situation (Gollwitzer, 1993, 1999). These are especially beneficial when they face obstacles or distractions and have a strong effect on goal achievement (Gollwitzer and Sheeran, 2006). Allowing oneself to get distracted from studying (procrastination) is a particular risk for students and a predictor of depression (Saddler and Sacks, 1993), decreased well-being, personal health (van Eerde and Klingsieck, 2018), and academic achievement (Steel et al., 2001; Kim and Seo, 2015). Imagine that someone wants to spend more time studying, but knows that his/her phone often distracts him/her from doing so for a longer period of time. The implementation intention could then be: ‘when I am going to study, I turn off my phone until I’ve spent at least 4 h studying.’

When students have trouble adjusting to the demands and context of tertiary education, they risk finding out about study issues when it’s too late. By the time the first exam results come in, it is hard to catch up, given that resits compete with the next exams that are scheduled (Schmidt et al., 2010). Self-efficacy and self-esteem moderately predict success, but the relationship works both ways (Lane et al., 2004; Honicke and Broadbent, 2016). In other words: past performance is also a predictor of self-efficacy and self-esteem. A weak or strong start thus reinforces the self-image and role of efficacy and esteem. When offered at the start of the study, the life-crafting intervention can kickstart self-regulated learning in time (Schippers and Ziegler, 2019).

Preventing these problems right on time, at the start of the study, could prevent a negative spiral. But apart from preventing these negative processes, this approach can also inspire a positive upward spiral. Walton (2014) reviewed an array of short, scalable psychological interventions that have large effects. He deems these wise because when they are offered to the right people at the right time, they can start a recursive process that reinforces itself. Reflective goal setting, according to participants who were followed over a longer period of time with a journal study (Travers et al., 2015) does just that, by bringing about engagement and experiences of flow. We thus propose that a life-crafting intervention right at the start of the study can start a recursive process. Life crafting shows great promise in terms of enhancing academic performance. Combining it with internet-based care could tackle three problems at the same time: the problems associated with adjusting to college life and self-discipline in studying, and mental health issues of students, as well as finding more meaning in life (Schippers and Ziegler, 2019). Below, we discuss findings related to internet-based care.

## Internet-Based Mental Health Care

Compared to online treatment, treating mental health issues with traditional face to face methods is costly. Internet-based or digital forms of mental health care can have the advantage of being scalable and, therefore, cost-effective. Several recent meta-analyses show that internet-based care can be as effective as traditional face to face therapy in treating mental health problems (Andersson et al., 2014; Carlbring et al., 2018). Because of its positive effects and its broad potential benefits, the Royal College of Psychiatrists in the United Kingdom advised universities to increase the availability of evidence-based online interventions for students (Royal College of Psychiatrists, 2011). Australia even has an official e-mental health strategy since 2006 (Meurk et al., 2016).

Although meta-analyses seem to show that online and analog therapeutic interventions have similar effects, some forms of online therapy and coaching have better adherence rates than others. We know, for instance, that (mental) health apps are generally used for a short period of time (about 2 weeks) before being abandoned (Baumel et al., 2019). While it may be that within this period, the beneficial effects are being delivered, it may also be desirable that people make use of such solutions for a longer period of time. Diefenbach and Niess (2015) found that 42% of users stop self-improvement technologies before significant progress is made. Lattie et al. (2019) showed that trials that lasted for 8 weeks showed the largest treatment effects in university student populations.

A recent meta-analysis aimed at online interventions in university contexts (Harrer et al., 2019) showed significant general effects of the interventions on stress (*g*^1^ = 0.20), depression (*g* = 0.20) and anxiety reduction (*g* = 0.27), role functioning (*g* = 0.41), and eating disorders (*g* = 0.52). Only four studies out of the 48 included trials measured outcomes on well-being. These four studies all used different scales for well-being (PWB, Core-OM, WEMWBS, and MHC). One of these studies (Kvillemo et al., 2016) used expressive writing exercises as an active control, to measure the effect of a mindfulness intervention, while expressive writing is known to improve well-being (Pennebaker et al., 1990; Pennebaker, 2004). If the latter study is excluded for this reason, a general significant effect of *g* = 0.25 on well-being can be found. Harrer et al. (2019) urge future researchers to study which interventions work best for specific types of students. They expect this ‘differentiation’ to further improve the effectiveness of the interventions.

Lattie et al. (2019) did a meta-analysis on internet-based care for university students, which included two trials that involved a chatbot (Fitzpatrick et al., 2017; Fulmer et al., 2018). Both trials reported high retention rates and significant positive effects on anxiety and depression. Other potential positive effects (e.g., performance or well-being) have not yet been studied, and chatbots have so far only been used to deliver CBT in a university context. However, these results seem promising. An intervention integrated with a chatbot is scalable, easily accessible, and adherence rates seem to be better than those for traditional online care.

Although the mental health and academic performance of students at the start of tertiary education are related, the literature and interventions aimed at preventing the interrelated problems are mostly separated. The first one aims at treating or preventing anxiety, depression, and other mental health problems among first-year students with online, digital interventions (Harrer et al., 2019; Lattie et al., 2019). This research and debate take place at the crossroads of clinical psychology, psychiatry, and information technology. Within this line of research, it is argued that going to college coincides with a decisive developmental phase into emerging adulthood (Arnett, 2006). The start of tertiary education coincides with a peak in the occurrence of mental health issues (Ibrahim et al., 2013; Auerbach et al., 2018; Bruffaerts et al., 2018). Online or digital treatment is (mainly) a more scalable and cost-efficient method to treat these difficulties (Ebert et al., 2018). The expected mechanism by which online therapy can help or aid is implied to be similar to the mechanisms that guide the effects of the ‘analog’ type of therapy (with a particular effective and often-used therapy: Cognitive Behavioral Therapy; Davies et al., 2014; Harrer et al., 2019). A potential unique beneficial quality of online treatment is anonymity, which was found to be related to more self-disclosure (Lucas et al., 2014, 2017). A downside seems to be higher attrition rates of participants (Baumel et al., 2019). Regrettably, students often do not feel inclined to volunteer to use these available treatments in time; only 20% of those who need it receive minimally adequate treatment (Auerbach et al., 2016), which is likely to result in worse clinical outcomes (Cheung et al., 2017). Research about the more durable and campuswide practical implementation of these treatments lacks in the current literature (Lattie et al., 2019). Chatbots that use AI and offer interactive therapy are at the forefront of the technological development within this field (Fitzpatrick et al., 2017; Fulmer et al., 2018), with more of the advantages of online therapy, and with a more personalized approach. These are applications that combine the benefits of anonymity with ‘rapport’ (Lucas et al., 2017).

The second line of research is aimed at improving the academic performance and well-being among students with goal-setting interventions. The data so far shows that goal setting can improve effort and direct effort to the right priorities (Locke and Latham, 2002). Goal setting helps students to allocate their time wisely and improve their academic performance and retention. Within this line of research, life crafting aims beyond just educational goals and strategies (Schippers and Ziegler, 2019). These integrative interventions stimulate students to formulate any type of goal, be they academic-, social- or health-related goals. Formulating goals and strategies to obtain the goals improves academic performance, regardless of whether the students formulated academic goals (Schippers et al., 2020). They argue that a potential spill-over effect is in place: If one formulates goals and does well in pursuing these within one field of life, this translates to positive effects in other domains. A meta-analysis from Klug and Maier (2015) shows that goal pursuit defined as progress instead of attainment, indeed increases (subjective) well-being. Together with Schippers et al.’s (2020) findings, this supports the hypothesis that formulating and strategizing about goals can be beneficial to both academic performance and well-being simultaneously.

We argue that the first line of research lacks the benefits of a more inclusive ‘positive’ approach that is aimed at all students through the curriculum of their university. This approach can be found in the second line of research. However, the second line of research, in turn, lacks the interactiveness and follow-up that online CBT therapy and chatbot technology provide. By combining these lines by integrating a goal-setting intervention with a chatbot and online CBT, we expect to activate three core mechanisms (right on time, inclusive approach, differentiated follow-up) that allow the integration of mental health chatbot- and life-crafting interventions to be worth more than the cumulation of its parts. In the following, we will specify how these mechanisms work within a chatbot platform and show concrete examples.

## Mental Health Care Chatbots

**FIGURE 1:**
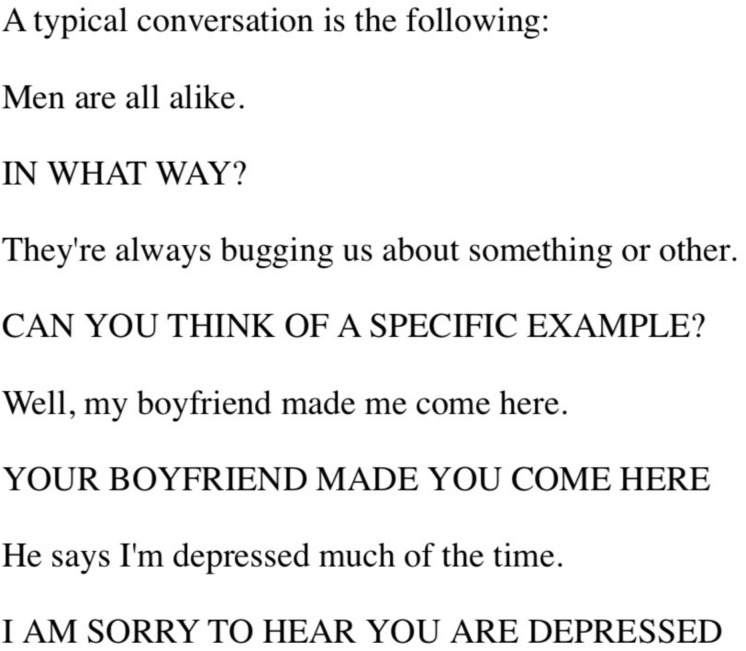
Conversation beween a human and chatbot [Reprinted with permission Weizenbaum (1966)].

Chatbots, also known as conversational agents, have gradually established themselves as companions to a multitude of modern devices. In the 1960s of the last century, Joseph Weizenbaum at MIT developed ELIZA (Weizenbaum, 1966), an early natural language processing computer program that simulated conversation and that is generally perceived as being the starting point in the development of conversational agents (Henderson, 2007; Jacques et al., 2019). Figure 1 shows a sample of a conversation between a human and ELIZA. Weizenbaum wanted to show how superficial the communication was between a human and a machine, but was surprised to find out that many individuals (including his secretary) would become emotionally attached to the program. They would even forget that they were conversing with a computer, and Weizenbaum’s secretary reportedly even asked him to leave the room from time to time in order to have a “real conversation” with the program (Bassett, 2019). The most famous script, DOCTOR, simulated a therapist that used the Rogerian way of conversing. Carl Rogers was a therapist who used non-directional questioning and often repeated back what a client said. The system would parrot phrases back, or ask to elaborate.

Since then, conversational systems have come a long way via intelligent assistants like Siri (Apple), Alexa (Amazon), and Cortana (Microsoft), social chatbots aimed at general conversation, and task-focused chatbots (Park et al., 2018; Shum et al., 2018; Jacques et al., 2019). Chatbots are spreading fast among websites and online services in functional areas such as customer service, marketing, entertainment, healthcare, and more. In order to improve the clarity of the discourse on chatbots, Braun and Matthes (2019) propose a framework via which chatbots can be categorized in terms of four characteristics beyond the functional application domain (see Table 1). Despite developments in speech recognition based on (a combination of) keywords, the development of *conversational skills* (e.g., actively keep a conversation going that feels natural) of AI has not improved in a similar pace (e.g., Park et al., 2018; Jacques et al., 2019).

**TABLE 1:** Chatbot classification framework (adapted from Braun and Matthes, 2019).

**Characteristic**	**Elements**	**Description**	
*I/O*	Voice	Speaking	The majority of current chatbots are text based.
	Text	Typing	
*Timing*	Synchronous	Real-time, direct interaction.	
	Asynchronous	Delayed interaction.	
*Flow*	Sequential	A specified order in which interaction is structured.	
	Dynamic	Information is processed in an arbitrary order.	
*Platform*	Messenger	Most current chatbots are connected to or build in a related functionality (like a website) and only a limited number are standalone.	
	Social media		
	Standalone		
*Understanding*	Notifications	Only sending messages.	
	Keywords	Automated word recognition.	
	Contextual	Include previous messages in the conversation thereby demonstrating understanding of context.	
	Personalized	Take information from external sources and/or previous conversations into account.	
	Autonomous	Independently communicate with humans and even other chatbots.	

Early chatbots depended on deterministic responses that are the result of a rule-based process, which results in chatbots that are perceived as less smart. The more commonly used machine learning techniques allow chatbots to go beyond fixed semantic responses. These techniques have the form of ‘supervised learning,’ using large datasets to train the chatbot which answers are appropriate and which are not; ‘unsupervised learning’ using Markov-chain based models; and ‘hybrid intelligence’ which combines both (c.f., Radziwill and Benton, 2017). The result has the form of highly complex decision trees consisting of if-then statements. Though this may sound like a simple principle, the fact that there is no fixed semantic model underlying the communication (i.e., an open conversation can be about anything, using any phrasing) leads to highly complex decision trees or even networks of decision trees. Training an algorithm capable of providing appropriate responses is complex and takes a lot of time, effort, and large quantities of training material and processing power (Lambert, 2018). Mass availability of personalized and autonomous chatbots, therefore, is expected only in 5–10 years (Weidauer, 2018).

## Design of a Mental Health-Oriented Chatbot for Education

The use of chatbots in education is still in its infancy. Though AI applications have been used to support learning for several decades, the overall application appears to be modest, but expectations regarding the future application and added value are high (Winkler and Söllner, 2018). A systematic review of 80 scientific papers on the use of chatbots in education (Winkler and Söllner, 2018) shows the main focus areas are health and well-being, language learning, providing feedback, and the support of metacognitive thinking, motivation, and self-efficacy. They found the usage of chatbot technology in support of learning to be influenced by individual student characteristics like personality traits, trust of and attitude toward technology, educational background, technological skills, and levels of self-efficacy and self-regulation. These findings match findings from the field of information systems research on technology acceptance (e.g., Davis, 1989; Venkatesh and Davis, 2000; Taherdoost, 2018).

The most prominent theories of technology acceptance include the Technology Acceptance Model (TAM; Davis, 1989; Venkatesh and Davis, 2000) and the Unified Theory of Acceptance and Use of Technology (UTAUT), which both are rooted in the Theory of Reasoned action (TRA; Ajzen, 1985) and the Theory of planned behavior (TPB; Fishbein and Ajzen, 1975). Research in this area has revealed a multitude of factors that contribute to technology acceptance, of which key predictors include the perceived ease of use and perceived usefulness of an application (Davis, 1989), playfulness (Moon and Kim, 2001), perceived presentation attractiveness (van der Heijden, 2004) and peer Influence (Chau and Hu, 2002). In the case of chatbots, perceived helpfulness has been found as an important predictor of user attitudes toward the use of technology (Zarouali et al., 2018). Technological applications in the area of education, personal development, and healthcare all share these characteristics underlying user acceptance.

The appeal of social chatbots in the area of mental health and well-being is large and primarily lies in their ability to make a social connection to users (Bickmore et al., 2005; Fitzpatrick et al., 2017; Shum et al., 2018). These chatbots show more promise than general mental health applications, through their potential to dynamically recognize emotion and to engage the user throughout conversations by showing appropriate responses (Shum et al., 2018). One of their main shortcomings, however, regards the so far inchoate ability to convincingly convey empathy to the user (Morris et al., 2018). In a clinical environment, for example, empathy has been identified as a key contributor toward better clinical outcomes as it lowers anxiety and distress, enhances satisfaction, and is directly related to higher patient enablement (Derksen et al., 2013). These effects are even more pronounced in the context of mental health interventions (Gateshill et al., 2011). Just as humans, non-human agents may struggle to express empathy (Morris et al., 2018). Still, research on mental health-oriented applications shows an overall user preference toward a chatbot compared to general non-conversational applications. Moreover, the use of non-conversational applications has been found to be abandoned after about 2 weeks by the majority of users (Baumel et al., 2019). By comparison, the adherence rate for a chatbot with a similar focus seems to be four times as long, as a chatbot can actively reach out and initiate communication with participants in a conversational way (Bickmore et al., 2005; Fulmer et al., 2018; Kamita et al., 2019). Expectations regarding the ability of chatbots to understand natural language and have meaningful natural conversations have not been met yet. However, as systems improve, the difference between humans and machine responses are expected to diminish (Jacques et al., 2019).

## Integrating the Life-Crafting Intervention With the Ai-Enhanced Mental Health Chatbot

Both life-crafting interventions and online mental health chatbot interventions have shown promising results when it comes to improving academic performance as well as mental health and subjective well-being. Integrating both can help in compensating for the downsides of each intervention. For instance, the life-crafting intervention is relatively static in its current form and could profit from the more interactional style from the chatbot. As mentioned before, a downside of the life-crafting intervention was that it did not respond to answers they gave or ask any follow-up questions whenever answers were brief. Writing more words corresponded with a larger effect of the treatment (Schippers et al., 2020), and stimulating students to write more, might make the intervention more effective. The life-crafting intervention starts in a browser and shows uniform texts, images, and videos that introduce uniform writing exercises (Schippers and Ziegler, 2019). Apart from demanding that students write at least one letter per question, there is no response to the brevity or content of what students write. Also, there is no differentiation in the intervention based on choices or texts from the students. All questions and follow up consisted of identical emails with goal setting diaries, which, according to students, did not feel personal and were soon experienced as spam.

The previously mentioned downsides of AI mental health chatbots are that students might be reluctant to volunteer for these interventions because of the stigma that is associated with mental health problems and because many students have trouble recognizing early symptoms of potentially serious mental health issues. Furthermore, these applications are mainly focused on alleviating mental health problems, and not on improving academic performance or eudaimonic well-being.

For these reasons, applying the chatbot to a more holistic approach (aimed not only at mental health problems but at life in general), in which the life-crafting intervention is integrated with an AI-enhanced mental health chatbot shows great promise. By combining a focus on life crafting, personal interactive coaching, and mental health, this approach is aimed at increasing general student academic performance and well-being, instead of merely focusing on potential problem areas. We suggest that all students receive this intervention at the beginning of their first year in tertiary education. That way, accessibility is large as all potential users will receive the intervention at the beginning of their first year. The opportunity to start using the chatbot at the start of the university studies has an added benefit toward early recognition and remediation of potential problems. The chatbot can play an important role in detecting (the development of) mental health problems as well as academic problems early on in the academic year. This way, we expect that the development of mental health problems can be prevented, or the student can receive additional online coaching on mental health issues by the chatbot early on, or the chatbot can refer the student to other sources of mental health coaching. Furthermore, the chatbot can also pro-actively seek contact with the student on the moments that the students’ stress level is expected to be on a high. For example, in the 3 weeks before a test week, the chatbot may check in with the student, inform whether the student is doing well, what learning goals have priority for the student at the moment, and ask if the student might need some help. We propose that this holistic, positive program aimed at what is most important for students combined with more differentiation could further enhance the user experience and improve its subsequent effects. A chatbot can thus be used not only in a curative way but also to detect problems early on and to prevent mental health issues from arising (Bendig et al., 2019; Schippers and Ziegler, 2019). Furthermore, the life-crafting intervention integrated into the chatbot can enhance academic performance and increase well-being for all participating students.

Within the chatbot platform, it is possible to differentiate between the needs of different students and thus offer a more personalized intervention. This personalization can be achieved in several ways. With regard to goal setting, self-regulated learning, and academic performance, students might report a wide range of issues. For example, some students might need help with the formulation or the prioritization of goals. Others might need help with regard to planning, monitoring, and time management, or ask for advice on how to learn in a better manner, for example with respect to learning strategies. With the chatbot, the set of effective self-regulatory processes for academic performance in higher education (De Bruijn-Smolders et al., 2016), based on the framework of self-regulatory processes as proposed by Sitzmann and Ely (2011) will be addressed with complementing evidence-based interventions. For example, with regard to planning, monitoring, and time management, students can be offered guidelines such as to study each day, to study the most difficult part first, and to use a to-do list when studying, and to make three kinds of planning, that is, for the day, the week, and for the long-term (for example until the test week; Gettinger and Seibert, 2002; Plant et al., 2005; Hattie, 2009). With respect to mental health, in line with the literature, we expect anxiety and depression to be most prevalent among the students (Auerbach et al., 2018). If students score high on the surveys on anxiety or depression, the chatbot will advise them to visit a student-advisor, and will advise them to follow cognitive behavioral therapy, online via the chatbot, or with an external professional. With respect to both, we want the chatbot to check in with the student at the right time (Walton, 2014); on the moments when we expect that students’ stress peaks will be on the rise, for example in the weeks before important exams, the chatbot will check-in with the student and offer customized coaching. Table 2 shows examples of what these conversations between a student and the chatbot could look like.

**TABLE 2:** Artificial enhanced life crafting, sample conversations chatbot and student.

**Life crafting intervention, four stages (Schippers and Ziegler, 2019)**		
**Stage 1: Finding Self-concordant passion or purpose (Sheldon and Houser-Marko, 2001)**		
**Chatbot:** What do you like to do? Write about this for approximately 2 min…	**Student:** Windsurfing	**Chatbot:** Hello <<student>> I notice that you have answered this question with one word. To use this intervention optimally, for your academic performance and well-being, it is important that you elaborate more on this. Can you tell me more about why you like windsurfing? Write about this for approximately 2 min…
**Stage 2: Mental contrasting (Oettingen, 2000, 2012) and formulating goal attainment plans (Schippers and Ziegler, 2019)**		
**Chatbot:** What do you admire in others…	**Student:** I admire fashion designers. Take for example Marc Jacobs. In a documentary I was inspired by his enormous drive, high energy level and positivity. His work really seemed to align with his passion. I hope I will also find what my passion is, my goal in life, and which job may fit with my passion optimally…	**Chatbot:** Thank you for your detailed answer, <<student>>. With the following questions, I aim to help you with finding your passion, your goal in life, and with making steps to attain your goal.
**Stage 3: Formulating and prioritizing students’ most important social, career, and health-related goals (Curran et al., 2015)**		
**Chatbot:** Now that you have your ideal future in mind, describe the goals that you can derive from this…	**Student:** Study more often (Academic goal), find a study-related job (Career-goal), Learn to deal with stress (Mental Health goal)…	**Chatbot:** Thank you <<student>>! Good to hear about your goals. I clearly see that you know what you want to achieve, which will be conducive for your academic success and well-being. However, unfortunately you will not be able to achieve all your goals at once. For this reason, can you rank them in order of importance?
**Stage 4: Designing implementation intentions [“If-then plans which help the student to set the right priorities in a complex situation (Gollwitzer, 1993, 1999)”]**		
**Chatbot:** Now you have specified your goals, make an if-then plan that will allow you to choose to do something that will help you to achieve your goal.	**Student:** I am not sure, can you give me an example?	**Chatbot:** Sure <<student>>. With respect to studying, an if-then plan might be: If I come home and am tempted to watch a drama series, then I will make sure that I first study for 2 h.
	**Student:** Ah, thank you <chatbot> When I am going to study, I turn off my phone until I’ve spent at least 4 h studying.	**Chatbot:** A solid if then-plan <<STUDENT>>, for studying more! Can you also formulate if-then plans for your other goals?
**Measurement scales well-being (anxiety, depression, self-efficacy, test anxiety,…)**		
**Chatbot:** This scale will help me get a better sense of your experience with anxiety OK? …	Student fills out General Anxiety Disorder 7-item scale (GAD7) (Spitzer et al., 2006).	**Possible answers chatbot:** This screening is not meant to be a diagnosis…. **Option 1:** Your responses indicate no or very few signs of anxiety. Keep in mind that minor ups and downs are normal. **Option 2:** However, your responses indicate some signs of anxiety. Would you like me to offer you cognitive behavioral therapy? This has helped other students in the past. **Option 3:** However, your responses indicate symptoms of anxiety. I recommend visiting one of the university student councilors, click here <<HYPERLINK>> to make an appointment. If you need immediate support you can call this number free of charge ≪CRISIS_NUMBER≫.
**Follow up: Stimulating and monitoring students goal progression via goal diaries^*a*^**		
**Chatbot:** OK <<STUDENT>>, let’s review the goals you worked on this week. Are you ready?	**Student:** Yes	**Chatbot:** What was the most important goal you wanted to accomplish last week?
	**Student:** Studying more	**Chatbot:** And, did you successfully complete what you were trying to accomplish?
	**Student:** No	**Chatbot:** What was the main reason for you that you were not able to study more?
	**Student:** I had not foreseen that certain tasks would take so much time. I did not have enough time to finish my study tasks this week.	**Chatbot:** Sorry to hear that <<STUDENT>>, do I understand it well that you had problems planning in your study tasks this week, and with your time management?
	**Student:** Yes	**Chatbot:** I want you to know that I can offer some coaching on this point. Are you interested to hear more from me about possible strategies to manage your time better, for studying more?
	**Student:** Yes	**Chatbot:** Alright <<STUDENT>> Let’s start (chatbot offers study interventions for better planning, monitoring, and time management) (e.g., see Gettinger and Seibert, 2002; Plant et al., 2005; Hattie, 2009).
**Chatbot pro-actively seeks contact with student. To begin with, the chatbot checks in on a weekly basis. Later on, the chatbot will check in more or less frequently, dependent on student’s needs.**		
**(1) Monitoring goal progression**		
**Chatbot:**<<STUDENT>> I want to check-in with you. Have you been making progress to your goals this week? To start with your health-related goal: did you exercise two times last week?	**Student:** No	**Chatbot:** What has gotten in the way?
	**Student:** I could not motivate myself to go to the gym, after I had had dinner at home.	**Chatbot:** I see, what can you do to make more progress with respect to your goal: exercise two times per week? Let me rephrase your if-then plan: “If I want to go to the gym the next day, then I will pack my sports bag the night before.” Was this if-then plan helpful with respect to your goal?
	**Student:** No, my if-then plan was not supportive for reaching my goal.	**Chatbot:** Can you think of an alternative if-then plan that will help you to accomplish your goal?
	**Student:** Yes. If I want to go to the gym, I will take my sports bag with me and go right after attending college.	**Chatbot:** Great! Well done <<STUDENT>>! Let’s see if your new if-then plan helps you better to reach your goal.
**(2) Emotional check-in**		
*Sample conversation chatbot and student*		
**Chatbot:** Hello <<STUDENT>>. How are you?	**Student:** I am worried, my grades are not so high, I do not know if I will pass this year.	**Chatbot:** I am sorry to hear that, <<STUDENT>>. In case you want to talk with someone about your situation, please feel welcome to contact one of the student advisers of ≪educational program≫. You can click here <<HYPERLINK>> to make an appointment right now.
**(3) Check-in right in time (e.g., 3 weeks before an exam period)**		
**Chatbot:** Hello <<STUDENT>>. Just checking in. How are you?	**Student:** I am very stressed. I am so busy preparing for my exams.	**Chatbot:** I am sorry to hear that, <<STUDENT>>. I can coach you on this, also I can offer you some strategies for dealing better with Stress. Sounds good?
**Student seeks contact with chatbot (24/7)**		
**Sample conversation chatbot and student**		
	**Student:** Hello <<chatbot>> I am feeling very lonely at the moment.	**Chatbot:** I am sorry to hear that <<STUDENT>>. Can you tell me more about why you are feeling lonely? …

*^*a*^As a follow up of the life-crafting intervention, the student receives six goal diaries to fill out, two-monthly, for monitoring and stimulating goal-progression.*

Moreover, within the life-crafting intervention, differentiation could also be applied. For example, according to Powers et al. (2005), implementation intentions, which is a part of goal setting, can be detrimental to students who score high on perfectionism. Some parts of the life-crafting intervention or even the complete intervention could not be beneficial to this particular subgroup. Short personality scales could be used before the onset of the intervention, to diversify the content of the intervention or even the complete intervention. A chatbot could start with an intake in which the student answers a survey on personality and well-being that allows the chatbot to offer a tailor-made program.

After the intake and a tailor made life-crafting intervention, the chatbot should remain readily available for regular cognitive behavioral therapy. But, as was also described by students who evaluated the life-crafting intervention, there should be a pro-active follow-up on the intervention. The chatbot will use the goals and strategies that the student has decided on to check-in on their progress. A chatbot can stimulate students to regularly reflect on, and remind them of, their goal progress with questions and personalized feedback. Schippers and Ziegler (2019) mention examples of questions that could be used for effective follow-up on the intervention: “Did I invest enough time into my goals? What could I do to improve this? Which smaller sub-goals could help me to achieve my objective? What obstacles do you face? What ways do you see to overcome them?” (pp. 11, 12). The chatbot can use cues in the answers of the students to offer the right type of strategies, for improved planning or combating procrastination for example.

## Conceptual Model

Some researchers state that merely having a goal already improves well-being (e.g., Klinger, 1977; Freund and Baltes, 2002). Gollwitzer and Brandstätter (1997) distinguish different phases in goal pursuit: predecisional (deciding about preferences between different goals or wishes), preactional (the initiation of goal directed actions), actional (successfully performing actions that bring a goal about) and postactional (evaluating results with the original intentions). Gollwitzer and Brandstätter state that it is to be expected that setting goals triggers predecisional and preactional goal pursuit. We predict that adding follow-up questioning and coaching via a chatbot can also improve the actional and postactional part of goal pursuit. In other words, setting goals initiates goal pursuit, but the follow-up through coaching from a chatbot can also improve the later phases of the pursuit of goals. Prior research has shown that goal pursuit, when conceptualized as goal progress instead of goal attainment in turn has an average effect of *r* = 0.45 on subjective well-being (Klug and Maier, 2015).

**FIGURE 2:**
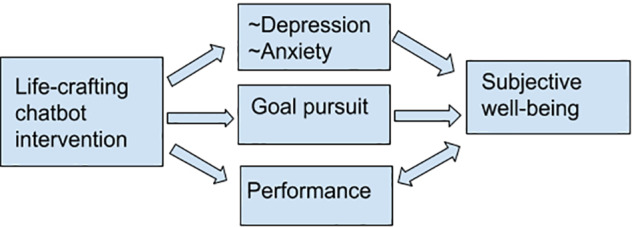
Conceptual model with expected mechanism of a life-crafting chatbot intervention.

We expect the low-threshold CBT therapy that the chatbot can offer based on intakes and scales that are included in the first part of the intervention to decrease anxiety and depression (Fitzpatrick et al., 2017; Fulmer et al., 2018). Including a large population of regular students in the treatment group might lead to results that differ from previous studies that only included students who volunteered to participate. Testing this is a necessary next step in the development of the literature. It is expected that goal progress influences SWB through an increase in positive affect, and the prevention of depression and anxiety improves SWB mainly through the negation of negative affect (see Figure 2). It is thus important to know how such a chatbot can be designed.

## Design Principles for a Life-Crafting Chatbot

Extant literature and experience have shown that the use of experimental or novel technologies is always associated with risks of low adoption. As Lattie et al. (2019) observe, digital mental health interventions, in particular, tend to fail due to acceptability, usability and feasibility issues. While in the previous parts we discussed potential issues and limitations that oftentimes plague such implementations, we stress the importance of the design philosophy before zooming in on the different design aspects themselves. Overall, human–computer interaction (HCI), in the context of every application, is a complex and dynamic experience that ever-evolves (as software gets updated). Naturally, the goal-setting intervention underlying the present study, as well as the chatbot used as the agent of delivery, also evolve based on the feedback and results received with each intake of students. The design principles, however, guiding the blueprint and evolution of the intervention should be grounded in suitable paradigms of HCI. In our cases, these are the design rationale (what user requirements does the platform intend to address? What are the reasons behind its particular features or the ones it doesn’t have? What are the trade-offs?) and usability engineering (*iterative development* based on usability specifications, *participatory design* by involving students in the development of the platform, *impact analysis* and overall cost-effectiveness evaluations) (Carroll, 1997). Following these two paradigms will allow us to address a number of issues related to the successful implementation of the intervention in a structured manner.

Current chatbot interventions in the university context can further improve their user-friendliness by (1) being more tailored to the intended users, (2) addressing issues that are most important to the users, (3) ensuring user privacy, (4) offering a trustworthy experience, and (5) offering aid in emergencies (Lattie et al., 2019). If user-friendliness is low, this will likely have a negative effect on the scalability, and durability of the intervention. Following a design rationale perspective, future research could address the first two concerns by identifying the specific needs of the target audience and their key issues that the intervention should be seeking to address. Following a usability engineering approach, we aim at fine-tuning and evolving the intervention in order to address its key shortcomings. This process involves focus groups and regular surveys over a prolonged period. To address the privacy and trust concerns of students, thorough regulation and transparency regarding the data management should be employed and effectively communicated to all participants.

The success of the intervention should be evaluated not only based on user satisfaction metrics but also by the overall user acceptance. The prolonged involvement of students with the chatbot is dependent on its user-friendliness. A chatbot is, by its nature, inherently more interactive and open then most used online interventions. Still, in the Fulmer et al. (2018) trial students did report that the chatbot biggest shortcomings included the chatbot not feeling natural (12/50), misunderstanding replies (11/50), not interactive enough (7/50) and impersonal (6/50). Extensive tests could make the chatbot more user-friendly.

If the chatbot is supposed to play a catalytic role in sustaining user-engagement throughout the intervention, key principles of HCI design need to be combined with key findings from the Technology Acceptance literature. As technology acceptance is concerned not with the unique experience and satisfaction but with the intention of users to change their ways and adopt a new technology in their routines, there needs to be focus on aspects of the design stimulating the key antecedents of acceptance, namely perceived usefulness/helpfulness, ease of use, and playfulness (Moon and Kim, 2001) as well as related antecedents of those such as technology readiness (optimism, innovativeness, discomfort, and insecurity) (Parasuraman, 2000) or technostress (Ayyagari et al., 2011). Developing such an integrated chatbot, with the use of modern technology combined with insights from positive psychology interventions such as life crafting, shows great potential in optimizing student well-being and (academic) achievement.

## Discussion

As many students struggle with academic underperformance and mental health problems during their transition to tertiary education, we sought to outline possible solutions that involve both the use of contemporary AI solutions and combine this with the latest insights from effective positive psychology interventions, specifically a promising life-crafting intervention. The advantages of such a solution are that it is scalable, has a low threshold, would contribute to early detection of academic or mental health problems, and would be interactive and personalized. We proposed an inclusive approach: all students could potentially benefit from the resulting intervention. Combining insights from two lines of research, namely the life-crafting (goal-setting) literature, and the literature on online mental health care, we proposed integrating a life-crafting intervention with a mental health chatbot could offer a solution for all students.

Our focus on scalability as an important criterion has to do with the fact universities are currently not able to cater to be 24/7 responsive to all the personal needs and mental health problems of their students. A chatbot is a scalable solution that is constantly available, because all students can individually take part in this intervention online. Only students with serious academic or mental health problems would be referred to the student advisor for further coaching or to, for example, psychologists. Our focus on a low threshold had to do with the fact that mental health problems bear a stigma that prevents many students from seeking help for these problems. Using a chatbot is anonymous, which is related to more self-disclosure and rapport (Lucas et al., 2014, 2017).

We proposed an inclusive approach, in which all students within a certain study program receive access to the intervention at the beginning of their first year of tertiary education. The main focus of the intervention is not mental health problems, but life crafting and setting personal goals, which can be beneficial to all students. Having this positive focus will probably also decrease the association with stigma on mental health problems. Only students who need it will also be able to receive mental health coaching via the chatbot. This touches another important criterion that we set for the intervention: differentiation. With a chatbot, it is possible to offer interactive and personalized coaching, based on the students’ individual needs. Moreover, the chatbot can also follow-up and interact with the students in later stages on what they have written in their intervention.

Finally, the chatbot can assist in early recognition of academic and mental health problems in two ways. First off, we expect that the life-crafting intervention integrated into the chatbot will make students more aware of their goals and potential obstacles. This will help them to set priorities for themselves, and may also encourage them to seek help for their problems in an early stage. Secondly, the chatbot itself can also recognize signals of academic or mental health problems, and offer in-app coaching (for mild problems) or refer to external help (for more severe problems) in early stages, if necessary. An additional advantage is that mental health chatbots often have higher adherence rates than other internet-based mental health care.

Future research should experimentally test the effects of interventions that combine insights from positive psychology which lend itself for curriculum wide implementation with the interactive potential of a chatbot. In line with Lattie et al. (2019) we propose that it would be of great value if these experiments were conducted in professional colleges or community colleges as well, besides research universities. It would also be highly recommended, to monitor technology acceptance, usability and implementation feasibility with validated scales. As Harrer et al. (2019) concluded, research on the effects of chatbots has so far not yet defined student subsets for which the intervention is most effective. A large scale experiment in which different student subsets are followed could, therefore, open up valuable new vistas which can further explore the added value of differentiation that a chatbot can offer.

In short, we expect that the proposed AI-enhanced life-crafting intervention will help students to overcome the difficulties they face when transitioning into tertiary education. We anticipate that it will increase students’ academic performance and decrease the development of mental health problems. Future studies will need to uncover the specific effects of this intervention. Ideally, this intervention will be able to optimize both student well-being and academic achievement.

## Author Contributions

MS and ID played the primary role in the conceptual conception of the manuscript. ED played a major role in structuring the manuscript. ED and ID together were principally responsible for the editing and revision process. MD, MS, and BG provided important intellectual feedback on several versions of the manuscript. ID, MD, BG, AA, ED, and MS contributed content for the original draft preparation of the manuscript. All authors agreed to all aspects of the manuscript and approved the final version.

## Conflict of Interest

The authors declare that the research was conducted in the absence of any commercial or financial relationships that could be construed as a potential conflict of interest.

## Footnotes

1 Hedges *g* was used as a common denominator in the meta-analysis of Harrer et al. (2019) because it adjusts for small sample size bias (Hedges and Olkin, 2014).
